# Long-term bowel dysfunction following low anterior resection

**DOI:** 10.1038/s41598-020-68900-8

**Published:** 2020-07-17

**Authors:** Audrius Dulskas, Povilas Kavaliauskas, Lukas Pilipavicius, Mantas Jodinskas, Martynas Mikalonis, Narimantas E. Samalavicius

**Affiliations:** 1 Department of Abdominal and General Surgery and Oncology, National Cancer Institute, 1 Santariskiu Str., 08406 Vilnius, Lithuania; 2 Faculty of Health Care, University of Applied Sciences, 45 Didlaukio Str., 08303 Vilnius, Lithuania; 3 Department of Abdominal and General Surgery and Oncology, National Cancer Institute, Clinic of Internal, Family Medicine and Oncology, Faculty of Medicine, Vilnius University, 1 Santariskiu Str., 08406 Vilnius, Lithuania; 4 Vilnius University Faculty of Medicine, Vilnius, Lithuania; 5 Surgical Department, Aalborg University Hospital, 18-22 Hobrovej, 9000 Aalborg, Denmark; 6 Department of Surgery, Klaipeda University Hospital, 41 Liepojos Str., 92288 Klaipeda, Lithuania; 7 Health Research and Innovation Science Center, Faculty of Health Sciences, Klaipeda University, Herkaus Manto street 84, Klaipeda, Lithuania

**Keywords:** Rectal cancer, Rectal cancer

## Abstract

Study aimed to assess long-term bowel function in patients who underwent low anterior resection for cancer five and more years ago. Patients who underwent low anterior resection for rectal cancer from 2010 to 2015 at National Cancer Institute were prospectively included in our study. They were interviewed using low anterior resection syndrome (LARS) score and Wexner questionnaire. We also assessed possible risk factors of postoperative bowel disorder. 150 patients were included in our study. Of them 125 (83.3%) were analysed. The median age at diagnosis was 62 years (40–79), and the average time of follow-up was 7.5 years (5–11). Overall, 58 (46.4%) patients had LARS, of them 33 (26.4%)—major LARS and 25 (20%)—minor LARS and 67 (53.6%) reported no LARS. Wexner score results were: normal in 43 (34.4%) patients, minor faecal incontinence—55 (44%), average faecal incontinence—18 (14.4%), complete faecal incontinence—9 (7.2%). 51 patients (40.8%) had tumour in the upper third rectum, 51 (40.8%)—in the middle and 23 (18.4%)—lower third. Preoperative (chemo)radiotherapy was the only significant risk factors for developing LARS in univariate analysis. Our study showed that only preoperative radiotherapy may be associated with more late problems in defecation after rectal cancer surgery.

**Trial registration**: NCT03920202.

## Introduction

Rectal cancer (RC) is a frequent and fatal disease with high incidence rate in developed countries, possibly because of differences in environment and diet^1^. The age standardized incidence rate among men and women in Europe, was 15–25/100.000 new cases of RC per year with a range of mortality from four to 10/100.000^2^. For the last almost 30 years, the gold standard treatment for RC is low anterior resection (LAR) with total mesorectal excision (TME) as described by *Heald*^3^.

Unfortunately, up to 80% of patients undergoing LAR will suffer of bowel dysfunction including faecal urgency, frequent bowel movements, tenesmus or so called Low Anterior Resection Syndrome (LARS)^4^. Simply it has been defined as “disordered bowel function after rectal resection, leading to a detriment in quality of life”^4^. Same year LARS score was developed^5^. This tool is easy to use and has been internationally and in Lithuania validated^6,7^. *Wexner* score is another tool for evaluation of faecal continence^8^. Recently Delphi consensus on LARS description was published. To meet the definition, a patient must have had an anterior resection (sphincter-preserving rectal resection) and experience at least 1 of suggested 8 symptoms that result in at least one of suggested 8 consequences^9^. The advantaged of the Delphi approach is that unlike most patient-reported outcome measures that were initially produced by expert clinician researchers who then consulted patient populations, the Delphi definition of LARS actively involved all major stakeholders, especially patients, early in the construction to ensure that the resulting tool was fit for purpose.

To our knowledge there are only five studies investigating long-term LARS following rectal surgery and influence it has on patients’ daily life using validated LARS score^10–14^. These studies showed that LARS was found in 47.5% to 90% of patients following rectal cancer surgery in long-term period.

Our aim was to evaluate late functional results of patients who underwent rectal resection for rectal cancer. This included calculating LARS and Wexner score and identifying possible risk factors of late postoperative bowel disorders.

## Materials and methods

### Study population and data collection

National Cancer Institute review board approval was acquired. All patients gave written informed consent for participation in our study.

The trial was registered in ClinicalTrials.gov—NCT03920202.

Data was collected prospectively of patients who received surgical treatment for RC between 2010 and 2015 at National Cancer institute in Vilnius, Lithuania. Between January to May 2020 all of the consented patients were interviewed face-to-face by same two interviewers in order to evaluate their bowel function outcomes using low anterior resection syndrome (LARS) score and Wexner score.

From 2010 through 2015, we prospectively identified a study population of 810 patients with biopsy-proven, rectal cancer without distant metastasis located up to 15 cm from anal verge and undergoing low anterior resection with partial (tumour in the upper 1/3 of the rectum—> 10 cm form the anal verge) or total mesorectal excision (if the tumour was in middle or lower 1/3 of the rectum—< 10 cm). Preoperative staging was performed based on a digital rectal examination, chest and abdominal computer tomography (CT) scan, pelvic magnetic resonance imaging (MRI) and colonoscopy with a biopsy. If patients was diagnosed with T3, 4N+ disease, they underwent long-course chemoradiotherapy (45 Gy given in 25 fractions over 5 weeks with adding in week one and week five 5-fluoropyrimidine-based chemotherapy), if it was < T3N0 or T2N+ tumour—upfront surgery. If the patient underwent preoperative chemoradiotherapy, low anterior resesction with anastomosis, he was a candidate for a protective ileostomy. All patients underwent open rectal resection with high inferior mesenteric artery (AMI) and inferior mesenteric vein (VMI) ligation with mobilization of splenic flexure. Circular anastomosis with end-to-end or side-to-end was performed. Of them 660 patients were excluded due to the exclusion criteria: dead patients (320), the patients underwent Hartman’s procedure or abdominoperineal excision (300), declined to participate (40). Of them, we included 150. 125 (83.3%) patients filled the questionnaires and were included in final analysis (20 were impossible to contact, five had permanent stoma). Possible risk factors: sex, age, tumour height (upper 1/3 vs middle 1/3 vs lower 1/3), type of anastomosis (side-to-end vs endo-to-end), postoperative course (with complications vs no complications), size of the tumour (according to T stage), presence of temporary ileostomy after the surgery (yes vs no), neoadjuvant treatment, nodular involvement, anastomotic diameter (according to circular stapler used) were assessed.

### Questionnaires

LARS score is a tool consisting of five items, which are as follows: incontinence due to flatus (score range from 0 to 7), incontinence due to liquid stools (score range from 0 to 3), frequency of bowel movements (score range from 0 to 5), clustering (score range from 0 to 11) and urgency (score range from 0 to 16). The severity of each item is calculated on a scale ranging from 0 to 42, with a score of 0–20 (no LARS), 21–29 (minor LARS) and 30–42 (major LARS).

Wexner score also consists of five items, which are as follows: solid incontinence (score range from 0 to 4), liquid incontinence (score range from 0 to 4), gas incontinence (score range from 0 to 4), pad wearing (score range from 0 to 4) and lifestyle alteration (score range from 0 to 4). The severity of each item was calculated on a scale ranging from 0 to 20. With a score of 0 (normal), 1–8 (minor faecal incontinence), 9–14 (average faecal incontinence) and 15–20 (complete faecal incontinence).

### Statistical analysis

For basic characteristics, we used descriptive statistics. Chi-square and student T-test were used to compare between groups or means. Univariate and multivariate logistic regression analysis.

was used to evaluate the odds ratio (OR) with the corresponding 95% confidence interval.

(CI). In order to examine age as a risk factor, patients were divided into two groups: group 1 (< = 62 years), consisting of 45 patients, and group 2 (> 62 years) as 62 was the median age. To compare two different questionnaires we used *Spearman* correlation index (rs). It can take values from + 1 to − 1. A rs of + 1 indicates a perfect association of ranks, a rs of zero indicates no association between ranks and a rs of − 1 indicates a perfect negative association of ranks. The closer rs is to zero, the weaker the association between the ranks.

Data was entered in Microsoft Office Excel 2013, calculated and analysed in IBM SPSS Statistics 23.0 (IBM Corp., Armonk, NY, USA). A *p* value < 0.05 was considered statistically significant.

### Conference presentation

The study was presented as a poster at International Society of Surgeons conference in Krakow, Poland August 11–15, 2019 and ESCP annual meeting Vienna, Austria September 25–27, 2019.

### Ethical approval

All procedures involving human participants were performed in accordance with the ethical standards of the institutional and/or national research committee and the 1964 Declaration of Helsinki and its later amendments or comparable ethical standards. This article does not contain any studies using animals.

## Results

**Figure 1:**
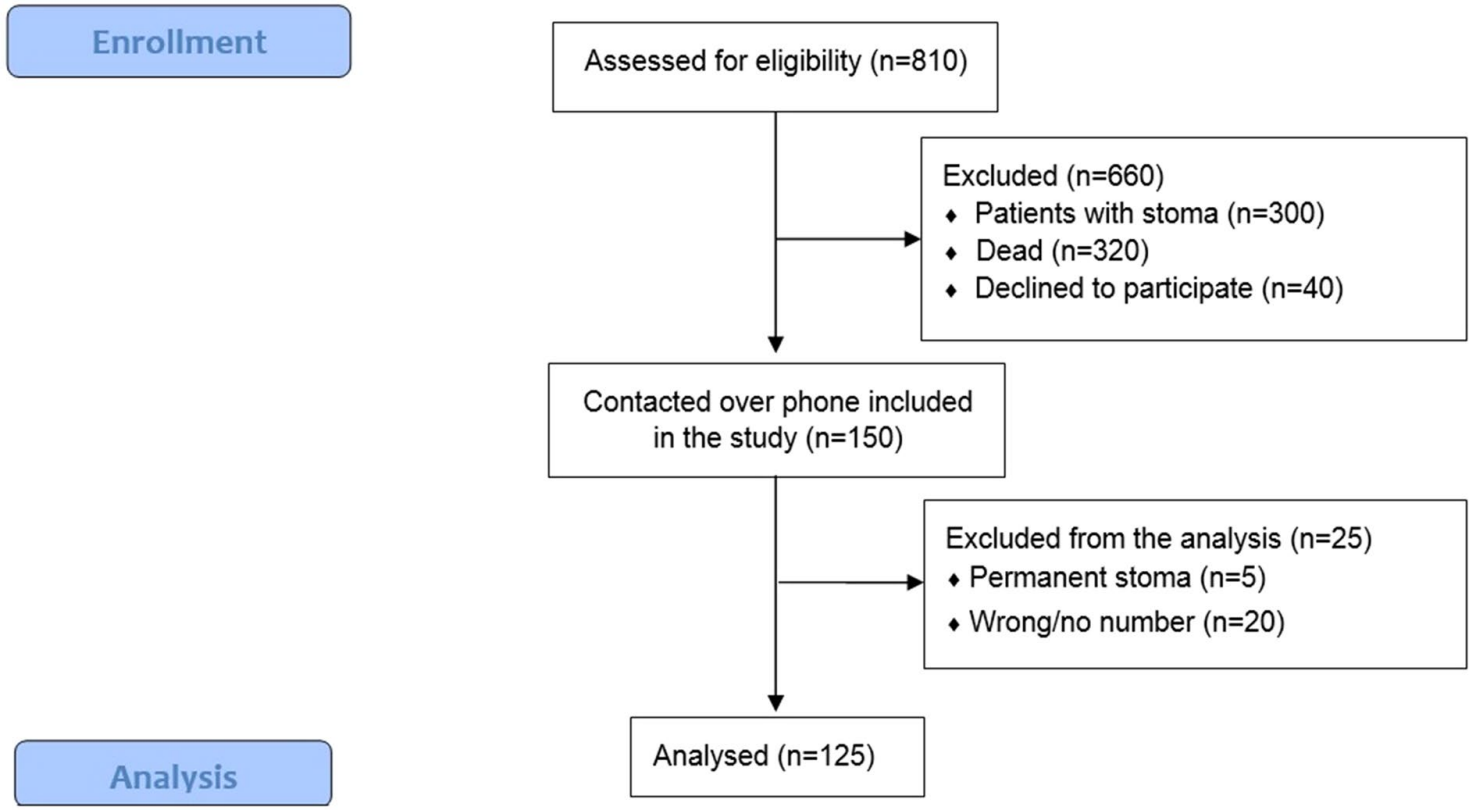
Consort flow diagram of the study.

Between 2010 and 2015, 810 patients underwent radical resection for rectal cancer at the National Cancer Institute in Vilnius. Of them 125 patients were included in our study (Fig. 1). Among this group, 64 (51.2%) were men and 61 (48.8%) were women (Table 1). The median age during operation was 69 years (45–88), and the average time of follow-up in the responders was 7.5 years (5–11). Overall, 58 (46.4%) patients had LARS, of them33 (26.4%)—major LARS and 25 (20%)—minor LARS and 67 (56.6%) reported no LARS. Wexner score results were normal in 43 (34.4%) patients, minor faecal incontinence—55 (44%), average faecal incontinence—18 (14.4%), complete faecal incontinence—9 (7.2%). 51 patients (40.8%) had tumour in the upper third rectum, 51 (40.8%)—in the middle part and 23 (19.40%)—lower third. 35 (28%) patients received preoperative radiotherapy and 13 patients (10.4%)—chemoradiotherapy. Further descriptive statistics are shown in Table 1.

**Table 1:** Characteristics of the patients included in our study.

Variables	Number, n (%)
No LARS	67 (53.65%)
Minor LARS	25 (20%)
Major LARS	33 (26.4%)
**Wexner score**	
Normal	43 (34.4%)
Minor faecal incontinence	55 (44%)
Average faecal incontinence	18 (14.4%)
Total faecal incontinence	9 (7.2%)
**Sex**	
Male	64 (51.2%)
Female	61 (48.8%)
**Neoadjuvant treatment**	
Yes	35 (28%)
No	90 (72%)
**Part of rectum affected**	
Upper 1/3	51 (40.8%)
Middle 1/3	51 (40.8%)
Lower 1/3	23 (18.4%)
**Preventive ileostomy**	
Yes	99 (79.2%)
No	26 (20.8%)
**Type of anastomosis**	
End to end	15 (12%)
Side to end	91 (72.8%)
No data	15 (12%)
**pTNM**	
Tis	4 (3.2%)
T1	16 (12.8%)
T2	31 (24.8%)
T3	62 (49.6%)
T4	7 (5.6%)
**Nodular involvement**	
Yes	35 (28%)
No	90 (72%)
**Postoperative complications (Clavien Dindo classification)**	
No complications	98 (78.4%)
I	3 (2.4%)
II	19 (15.2%)
IIIA	1 (0.8%)
IIIB	2 (1.6%)
IVA	1 (0.8%)
IVB	1 (0.8%)

Univariate analysis showed that only neoadjuvant treatment was associated with Major LARS prevalence (Table 2). Univariate analysis for average and total faecal incontinence did not show any significant risk factors.

**Table 2:** Univariate analysis of risk factors for major low anterior resection syndrome (LARS).

Factor	ODDS ration	*P*	95% CI
**Gender**			
Male	1	0.442	0.62–3.04
Female	1.37		
**Part of rectum affected**			
Upper 1/3	0.4		0.13–1.23
Middle 1/3	0.86	0.165	
Lower 1/3	1		
**Age**			
< 62 years	0.52	0.117	0.23–1.18
> 62 years	1		
**Overal postoperative complications**			
No	0.97	0.95	0.37–2.56
Yes	1		
**Overall comorbidities**			
No	1.3	0.522	0.58–2.9
Yes	1		
**Type of anastomosis**			
End to end	2.37		0.64–8.8
No data	2.89	0.165	0.6–13.6
Side to end	1		
**pTNM**			
Tis	0.5		0.23–11.1
T1	0.5		0.45–5.5
T2	0.35	0.89	0.37–3.31
T3	0.48		0.54–4.3
T4	1		
**Nodular involvement**			
Yes	1.74	0.21	0.73–4.02
No	1		
**Preventive ileostomy**			
Yes	1	0.065	0.93–11.95
No	3.33		
**Neoadjuvant therapy**			
Yes	2.82	0.016	1.22–6.53
No	1		

Spearman correlation showed a strong relationship between LARS score and Wexner score—r = 0,723 (*p* < 0.000).

## Discussion

In this study, we found that less than half of the patients (46.4%) reported LARS symptoms and 26.4% of them had major LARS. Three-fourths (76.4%) had no or had only minor faecal incontinence according to Wexner score. The average time of follow-up was 7.5 years, so in our opinion results are quite satisfactory. However, one-fourth (26.4%) of the patients had major LARS score and nine (7.2%) patients had complete faecal incontinence according to Wexner score. In searching for possible risk factors associated with higher LARS and Wexner scores, we identified that patients who received preoperative chemoradiotherapy, were more likely to have a higher LARS score. Relatively lower prevalence of LARS compared to other studies might be related to lower number of patients undergoing preoperative chemoradiotherapy (90–72%) and higher number of patients with upper third rectal cancer (51–40.8%).

Comparing our results to previous studies (Table 3) worst results are seen in Beppu et al. study^10^. Differently from us authors included only patients with low rectal cancer after chemoradiotherapy. 78 of 87 (90%) patients experienced LARS. Chen et al. in their study showed that major LARS was present in 46% of patients 15 years following the surgery, and major LARS was associated with reduced quality of life^11^. Authors found only two risk factors for major LARS: preoperative chemoradiotherapy and age < 75 years. Meanwhile Sturiale et al. followed the patients for average 13.7 years. They reported LARS in 44 patients (47.5%), with major manifestations in 19 patients (20.5%), and minor symptoms in 25 patients (27%). Age more than 70 years, tumour distance from the external anal verge, neoadjuvant treatment, and interval time of closing stoma were independent prognostic factors of functional disorders after surgery^12^. Other possible risk factors, such as gender, and interestingly tumour height or presence of the ileostomy did not reveal any significant impact on LARS or Wexner scores. Differently from our study, Gadan et al. did show the long-term negative effect of ileostomy formation on bowel function^13^. Similarly to our study, Pienowski et al. found in a long-term preoperative chemoradiotherapy, younger age and low level of the tumour were the risk factors of having LARS, and having major LARS^14^.

**Table 3:** Previous studies on long-term low anterior resection syndrome (LARS) incidence.

Refernces	Number of patients included	Institution	Incidence of long-term LARS n, (%)	Time following the surgery (y)
Beppu et al.^10^	87	Single centre	78 (90)	6.5
Chen et al.^11^	241	Multi centre	164 (68)	14.6
Sturiale et al.^12^	93	Two centres	44 (47.5)	13.7
Gadan et al.^13^	87	Multi centre	63 (72)	12
Pieniowski et al.^14^	282	Multi centre	237 (73)	> 5
Our results	125	Single centre	30 (44.77)	7.5

It might seem that studies assessing the long-term bowel dysfunction is a very new insight. Actually, we purposely have discussed only the studies with certain tools used to assess bowel dysfunction. All these studies used LARS score to assess the LARS following the surgery. Some historical studies from late nineties also assessed bowel function changes following rectal cancer surgery^15–18^. Hida et al*. from* Japan conducted a prospective study including 46 patients who underwent J-pouch reconstruction (J-group) and 49 patients who underwent straight anastomosis (S-group) after LAR for rectal cancer^15^. The assessed the bowel function using a 17-item questionnaire and mano-volumetry. They found that the number of bowel movements during the day (≥ 5, 4.3 vs. 29.2 percent; *P* = 0.028) and at night (> 1/week, 4.3 vs. 33.3 percent; *P* = 0.013) and urgency (4.3 vs. 33.3 percent; *P* = 0.013) and soiling (21.7 vs. 50.0 percent; *P* = 0.043) were less in the J-group than in the S-group. Dehni et al. also assessed long-term bowel function comparing same two techniques for anastomosis (J-pouch vs straight anastomosis) in 81 patients^16^. The average time following the surgery was 5 years. Patients with colonic J-pouch-anal anastomosis had better function in terms of frequency of defecation (1.57 + 1 vs. 2.79 ±  1; *P* = 0.001) and presence of irregular transit or stool "clustering" (30 vs. 71 percent; *P* = 0.003). The patients with J-pouch also had less repeative need to defecate again within one hour. However, it is impossible to assess percentage of bowel dysfunction from the whole number of patients. It is seen that up to 70–80% of patients included in the study had some degree of bowel dysfunction. Differently from studies mentioned above, Floodeen et al. assessed a total of 123 patients who underwent low anterior resection and had protective ileostomy (stoma group) and had no ileostomy (no-stoma group)^17^. They contacted the patients 5 years following the surgery and filled the bowel function questionnaire with 10 questions (65 in the no-stoma group and 58 in the stoma group). Daytime stool frequency at 5-year follow-up was a median 2.5 in the no-stoma group and 3.0 in the stoma group, and one third of the patients in both groups could not defer defecation for ≥ 15 min. Regarding the need for medication to open the bowel, evacuation difficulties, fragmentation of bowel movements, and incontinence, there were no differences between the 2 groups. Wagman et al. assessed 35 patients undergoing radiation and surgery with coloanal anastomosis using simple scale for long-term sphincter function^18^. The median follow-up was 56 months [range 4–121 months]. Sphincter function was excellent in 59%, good in 26%, fair in 15%, and none had poor function. Therefore, 85% (23) of the 27 evaluable patients had good or excellent sphincter function. All patients had complete continence of solid stool. Although minor soiling (40%) and difficulty with evacuation were common (50%), these problems were well tolerated.

These studies obviously gives information on long-term bowel function, but lack of using same validated questionnaires is a major limitation. Another drawback is the inclusion of studies with the specific primary outcomes (comparative studies of two techniques) and not describing a real world data.

We have to take into account that 25 patients from the primary list were excluded due to various reasons which lead to decreased number of patients in our study (from, the response rate was %) and this could be seen as one of the drawbacks of this study. In addition, a correlation between postoperative LARS and Wexner scores was found. It seems LARS and Wexner scores can be used together to evaluate the patients more broadly than with LARS alone.

Our studies has some strengths. First, it uses two most often met scores for bowel function assessment in patients following rectal surgery and assesses the correlation. Secondly, we have more than 120 patients—highest number from the single centre so far. Another strength of our study—we are presenting the highest number of patients included from the single institution base and not from other clinical trial^11,13^—the real world data presented. The greatest strength of this study is the response rate of more than 90% in those who the questionnaires was shown to.

Our study has other limitations also. First, small sample size and the risk of possible type 2 error—there is always a possibility that those patients who were not included in the study died or did not want to participate had worse functional outcome and only those with better function answered the questions. Second, we did not perform the longitudinal assessment of bowel symptoms within the period. Lastly, our study was restricted to the patients from single centre, including only small number from all treated rectal cancer patients. However, our institution is the tertiary referral cancer centre and performs more than 40% of all LARs in Lithuania.

To conclude, our study shows that LARS symptoms last many years after the surgery. Furthermore, our results show that bowel dysfunction, is a chronic condition and the patient will need support and treatment over a long time. This knowledge is important in discussions with patients (especially older patients with already affected bowel function) with severe LARS symptoms who may prefer a permanent stoma. Further studies are needed to increase the prevention of LARS, improve treatment and to find best support of patients with LARS.

## References

[1] Jemal A, Bray F, Center MM (2011). Global cancer statistics. CA Cancer J Clin..

[2] Glimelius B, Tiret E, Cervantes A (2013). Rectal cancer: ESMO clinical practice guidelines for diagnosis, treatment and follow-up. Ann Oncol..

[3] Heald RJ (1979). A new approach to rectal cancer. Br J Hosp Med..

[4] Bryant CL, Lunniss PJ, Knowles CH, Thaha MA, Chan CL (2012). Anterior resection syndrome. Lancet Oncol..

[5] Emmertsen KJ, Laurberg S (2012). Low anterior resection syndrome score:development and validation of a symptom-based scoring system for bowel dysfunction after low anterior resection for rectal cancer. Ann Surg..

[6] Juul T, Ahlberg M, Biondo S, Emmertsen KJ, Espin E, Jimenez LM (2014). International validation of the low anterior resection syndrome score. Ann Surg..

[7] Samalavicius NE, Dulskas A, Lasinskas M, Smailyte G (2016). Validity and reliability of a Lithuanian version of low anterior resection syndrome score. Tech Coloproctol..

[8] Jorge JM, Wexner SD (1993). Etiology and management of fecal incontinence. Dis Colon Rectum..

[9] Keane C, Fearnhead NS, Bordeianou LG, Christensen P, Basany EE, Laurberg S, Mellgren A, Messick C, Orangio GR, Verjee A, Wing K, Bissett I, LARS International Collaborative Group (2020). International consensus definition of low anterior resection syndrome. Dis. Colon Rectum..

[10] Beppu N, Kimura H, Matsubara N, Tomita N, Yanagi H, Yamanaka N (2016). Long-term functional outcomes of total mesorectal excision following chemoradiotherapy for lower rectal cancer: stapled anastomosis versus intersphincteric resection. Dig Surg..

[11] Chen TY, Wiltink LM, Nout RA, Meershoek-Klein Kranenbarg E, Laurberg S, Marijnen CA, van de Velde CJ (2015). Bowel function 14 years after preoperative short-course radiotherapy and total mesorectal excision for rectal cancer: report of a multicenter randomized trial. Clin. Colorectal Cancer..

[12] Sturiale A, Martellucci J, Zurli L, Vaccaro C, Brusciano L, Limongelli P, Docimo L, Valeri A (2017). Long-term functional follow-up after anterior rectal resection for cancer. Int. J. Colorectal. Dis..

[13] Gadan S, Floodeen H, Lindgren R, Matthiessen P (2017). Does a defunctioning stoma impair anorectal function after low anterior resection of the rectum for cancer? A 12-year follow-up of a randomized multicenter trial. Dis. Colon Rectum..

[14] Pieniowski EHA, Palmer GJ, Juul T, Lagergren P, Johar A, Emmertsen KJ, Nordenvall C, Abraham-Nordling M (2019). Low anterior resection syndrome and quality of life after sphincter-sparing rectal cancer surgery: a long-term longitudinal follow-up. Dis. Colon Rectum..

[15] Hida J, Yoshifuji T, Tokoro T (2014). Comparison of long-term functional results of colonic J-pouch and straight anastomosis after low anterior resection for rectal cancer: a five-year follow-up. Dis. Colon Rectum..

[16] Dehni N, Tiret E, Singland JD (1998). Long-term functional outcome after low anterior resection: comparison of low colorectal anastomosis and colonic J-pouch-anal anastomosis. Dis. Colon Rectum..

[17] Floodeen H, Lindgren R, Hallböök O, Matthiessen P (2014). Evaluation of long-term anorectal function after low anterior resection: a 5-year follow-up of a randomized multicenter trial. Dis. Colon Rectum..

[18] Wagman R, Minsky BD, Cohen AM, Guillem JG, Paty PP (1998). Sphincter preservation in rectal cancer with preoperative radiation therapy and coloanal anastomosis: long term follow-up. Int. J. Radiat. Oncol. Biol. Phys..

